# A rare lumbar pyogenic spondylodiscitis caused by staphylococcus caprae with initial misdiagnosis: case report and literature review

**DOI:** 10.1186/s12893-020-00860-2

**Published:** 2020-09-14

**Authors:** Zihan Fan, Yong Yang, Dong Li, Qi Fei

**Affiliations:** Department of Orthopedics, Beijing Friendship Hospital, Capital Medical University, No.95, Yong’an Road, Xicheng District, Beijing, 100050 China

**Keywords:** Pyogenic lumbar spondylodiscitis, Staphylococcus caprae, Vertebral needle biopsy, Case report

## Abstract

**Background:**

Staphylococcus caprae (Sc) is an uncommon causative organism for human. Lumbar pyogenic spondylodiscitis (LPS) of Sc is extremely rare and only a few cases have been reported. As far as we know, there is no specific literature on the diagnosis and treatment for LPS of Sc with L5 nerve root irritation.

**Case presentation:**

A 65-year-old male patient complained of chronic low back pain for 10 years, acute worsening with radiating pain to left lower extremity over a month. Physical examination revealed tenderness point on his low back, 3/5 dorsiflexor strength in his left 1st toe and decreased sensation of pin prick over the left lateral shank and medial dorsal foot. The individual was initially misdiagnosed with lumbar disc herniation (LDH) without further examination in outpatient, which was then found to be LPS of Sc with L5 nerve root irritation after admission to our hospital. Magnetic resonance images (MRI) of lumbar spine exhibited inflammation signal at L4-L5 level of the vertebral body and disc with hypointense on T1-weighted images (T1-WI) and hyperintense on T2-weighted images (T2-WI). The causative organism was confirmed by the culture of irrigation fluid obtained from L5 vertebrae by needle puncture. After systemic conservative treatment including using sensitive antimicrobial agents and immobilization, the rare infection was finally cured. The patient also showed a satisfactory recovery during the 36-month follow-up period.

**Conclusions:**

Confirming the diagnosis and identifying the causative organism as soon as possible is the key point for the treatment of LPS. LPS of Sc causing nerve root irritation is rare but curable with early diagnosis and proper therapy. The culture of irrigation fluid obtained from vertebrae by needle puncture may be an effective and sensitive attempt for potential infection of spine to identify the causative organism at early stage of the disease.

## Background

Staphylococcus caprae (Sc) was firstly reported by Devriese et al. in 1983 based on a strain which was the main species isolated from goat’s milk [1]. The organism has been well described in veterinary medicine [2–4]. As a kind of coagulase-negative species of Staphylococcus, Sc is considered to be a commensal organism of the skin and mammary glands of goats. However, this kind of Staphylococcus may also typically colonize human skin, nose and nails. It has been reported in several cases as a human pathogen causing peritonitis, meningitis, urinary tract infections, endocarditis, endophthalmitis, prosthetic joint infections, recurrent sepsis, bacteremia and osteomyelitis [5–9]. Many risk factors for the infection of Sc have begun to emerge including immunosuppression, diabetes, chronic renal failure, obesity, open or traumatic fractures and contact with sheep or goats [6]. In recent years, Sc has also been reported as a human hospital-acquired pathogen, mostly implicated in bone and joint infection after orthopedic operations [5, 10, 11]. But there are rare cases for infection of Sc occurring in native bone and joints without orthopedic prostheses. Lumbar pyogenic spondylodiscitis (LPS) is an uncommon lumbar infection involving intervertebral disks and adjacent vertebral bodies. The most well-known causative microorganism of LPS is *Staphylococcus aureus* (Sa). Hence, LPS caused by Sc is more rarely reported. At the early stage of the disease, LPS of Sc with L5 nerve root irritation is quite familiar with lumbar disc herniation (LDH), but the therapy of these two kinds of diseases is totally different. Therefore, differential diagnosis between LPS of Sc and LDH must be seriously concerned, as misdiagnosis and delayed treatment may lead to an irreversible damage in neurologic function even physical disability for the patient. Here, we present the clinical, bacteriological, pathological, radiological findings, and treatment outcomes in a new case of LPS of Sc with L5 nerve root irritation.

## Case presentation

A 65-year-old male patient was referred to our hospital because of chronic low back pain for 10 years, acute worsening with radiating pain to left lower extremity over a month. The patient had a chronic low back pain for 10 years. He was diagnosed with LDH and lumbar fasciitis and received conservative treatment in local hospital. The back pain was acute worsening with radiating pain and numbness to the left shank and 1st toe over a month. The patient had experienced Traditional Chinese Medicine (TCM) acupuncture treatment on lower back for five times in the past 1 month before admitting to our hospital. The patient told the longest needles were about 7–8 cm and he could feel the needle hit on the bone during the process of acupuncture. He had a history of hypertension which was controlled well with medication for 10 years without history of any kinds of infection. There was no contact history of cows, goats or raw milk as well. The patient had a 30 years history of smoking, about ten cigarettes per day. There was no history of tuberculosis  (TB), alcohol abuse or illicit drug use as well as family history of other diseases for him. On physical examination, the patient had no fever or loss of weight, and his vital signs were stable. However, he had tenderness points on his low back, and had 3/5 dorsiflexor strength in his left 1st toe. Decreased sensation of pin prick over left lateral shank and medial dorsal foot was documented. The straight leg raising test was positive in this patient’s bilateral lower extremities. The Visual Analogue Score (VAS) was 7.

**Fig. 1:**
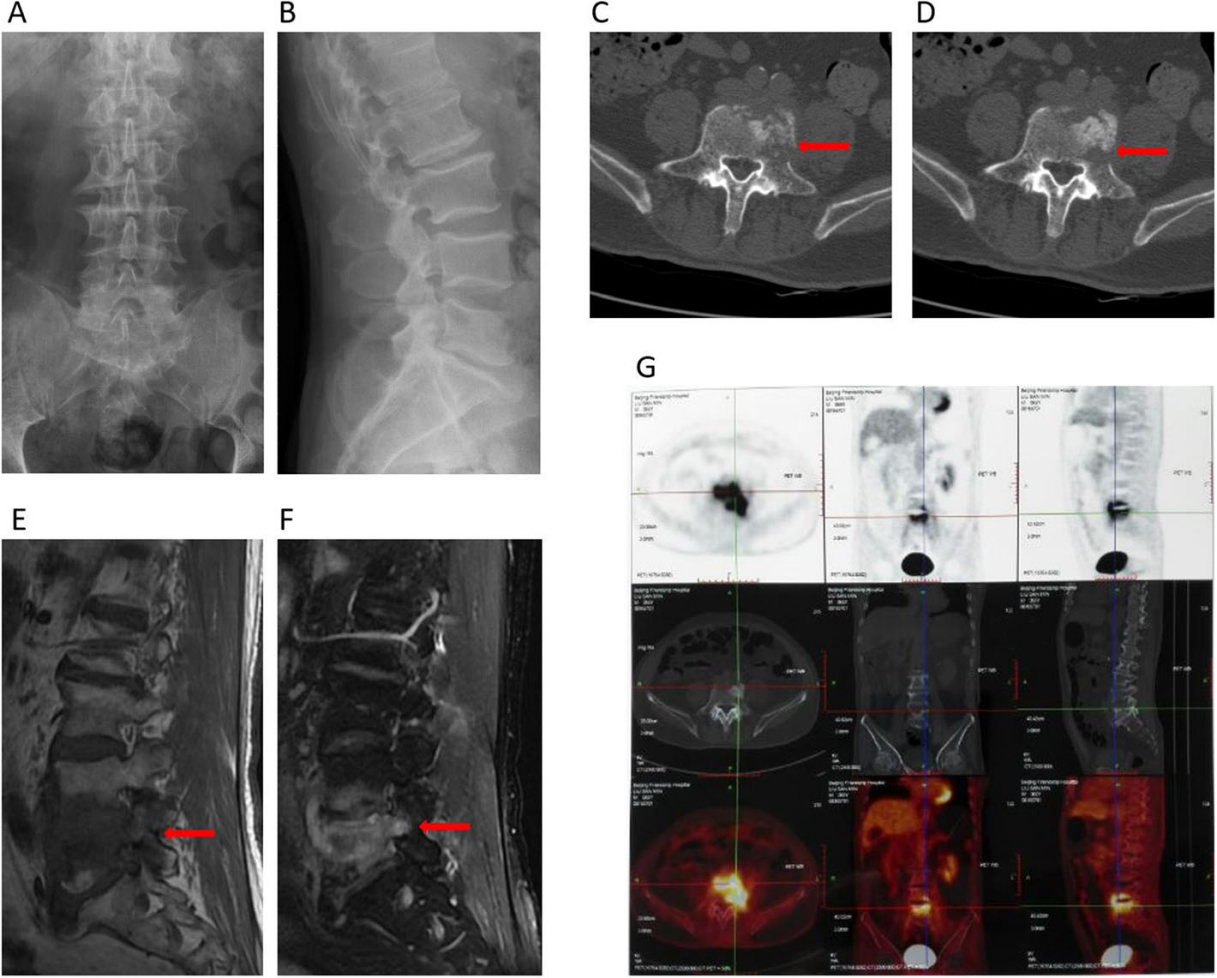
Radiological findings before treatment. Notes: (**a** and **b**) X-ray before treatment showed degeneration of lumbar spine, hyperplasia of vertebrae, no significant narrowing of L4–5 inter-vertebral space**.** (**c** and **d**) CT showed vertebral worm-eaten-like damages (arrow), the formation of sequestrum, hyperplasia of vertebrae. (**e** and **f**) MRI showed hypointense on T1-WI and heterogeneous hyperintense on T2-WI (arrow) in L4–5 level of vertebrae and disc. (**g**) PET-CT showed the significant uptake of the radioactive FDG in L4–5 level, high suspicion of infection

Laboratory examinations indicated a normal white blood cell count of 7.6 K per mm^3^, erythrocyte sedimentation rate of 69 mm h^− 1^, and C-reactive protein of 47 mg L^− 1^. Lumbar X-ray showed degeneration of lumbar spine, hyperplasia of vertebrae, no significant narrowing of inter-vertebral space at L4-L5 level (Fig. 1a and b). When the patient firstly arrived at the outpatient department, he was diagnosed as LDH due to low back pain radiating to left lower extremity. After admission to inpatient department, further examination revealed more details, which were not detected before, leading to a new and more accurate result. The rose bengal plate tests, anti-mycobacterium tuberculosis antibody and PPD were tested, showing negative results. Computed tomography (CT) scan revealed vertebral worm-eaten-like damages, the formation of sequestrum, hyperplasia of vertebrae at L4-L5 level (Fig. 1c and d). Magnetic Resonance Imaging (MRI) of the lumbar spine revealed inflammation like signal at L4–L5 level of the vertebral body and disc which exhibited hypointense on T1-weighted images and hyperintense on T2-weighted images (Fig. 1e and f). To exclude neoplastic disease, PET-CT was also performed which showed the significant uptake of the radioactive FDG, high suspicion of infection (Fig. 1g). Iliac bone biopsy was also implemented by hematologist for the possibility of hematological disease, pathological report of which showed there was no finding of malignant tumor cells.

**Fig. 2:**
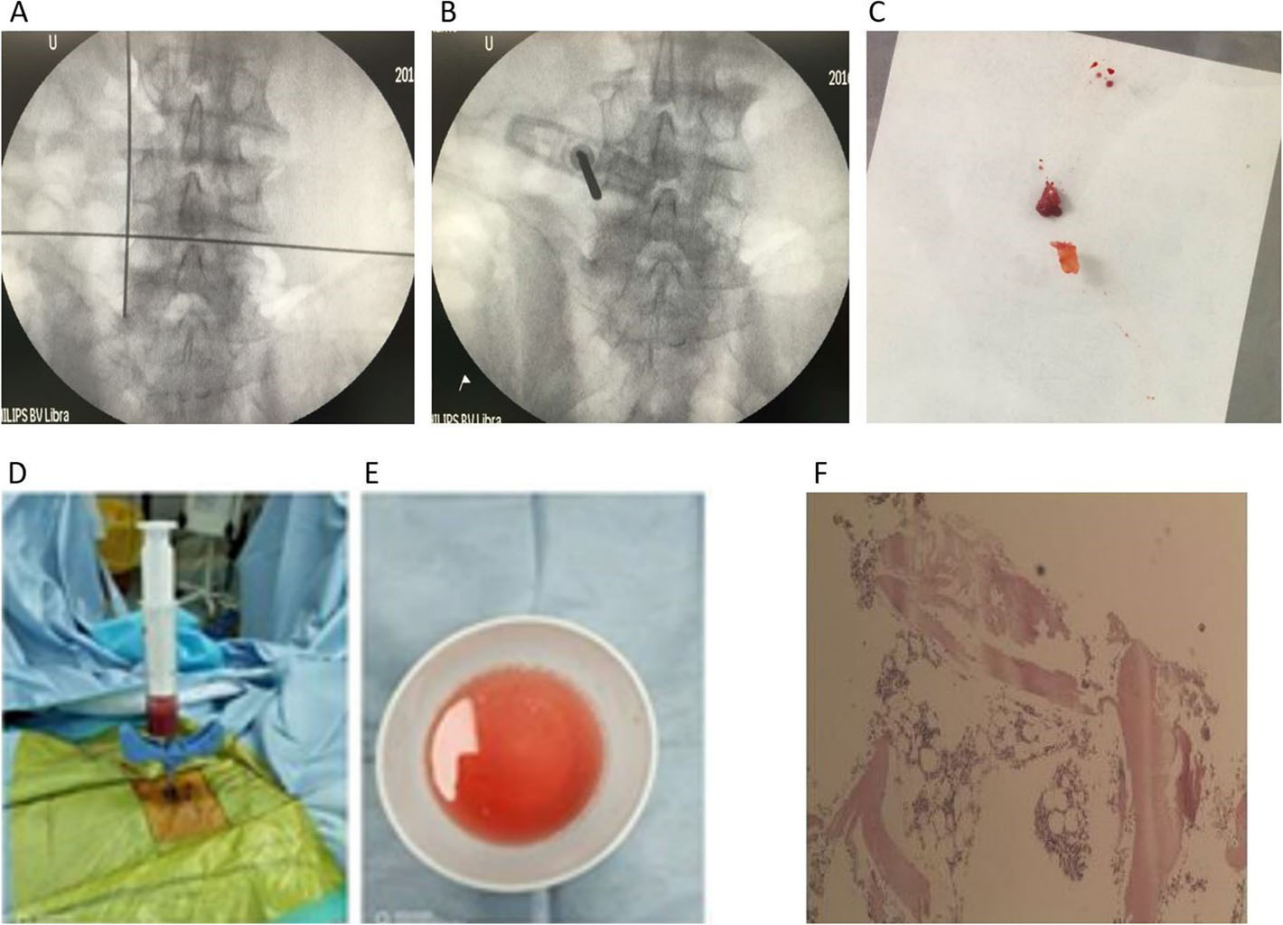
Pictures during the operation and pathological report of L5 bony tissue biopsy. Notes: (**a** and **b**) X-ray pictures of fluoroscopy during operation, L5 vertebrae was punctured from left pedicle by needle. (**c**) bony tissue was obtained from L5. (**d** and **e**) Sterile saline solution was irrigated through needle into L5 by syringe and collected for aerobic and anaerobic bacteria culture and drug susceptibility test. (**f**) Pathological report showed diffused distribution of plenty of lymphocytes without the gathering of epithelioid cell granulomas, granular necrosis and Langerhans giant cells

To confirm the diagnosis, fluoroscopy guidance-guided needle (Dragon crown, Shandong, China) puncture of the L5 vertebral body was performed (Fig. 2a and b). We decided to choose the left transpedicular approach as the puncture path, because the left side of the vertebral body was mostly affected (Fig. 1c and d). On the anterior–posterior (AP) view of fluoroscopy, the tip of needle was inserted on the superior-lateral quadrant of the pedicle. The trajectory of the needle passed through the soft tissues and left pedicle into the vertebral body. The biopsy forceps (JZ classic, Shanghai, China) were applied to obtain the bony tissue for further examination (Fig. 2c). The sterile saline solution irrigated through needle into L5 by syringe was collected as well for cytology, culture and drug susceptibility test (Fig. 2d and e). In order to avoid the spread of the infection, we tried to minimize the stab incision within 4 mm and executed asepsis strictly. Pathological report of L5 needle biopsy showed that diffused distribution of plenty of lymphocytes without any granulomatous reaction characterized as the gathering of epithelioid cell granulomas, granular necrosis and Langerhans giant cells (Fig. 2f). The staining and smear microscopy of acid-fast bacilli (AFB) was also negative. Meanwhile, the bacterial culture of fluid irrigated from L5 was growing Sc. The Final diagnosis was LPS of Sc.

Antibiotic susceptibility test revealed that the top two kinds of the most sensitive antimicrobial agents were vancomycin and linezolid (Table 1). According to the result of drug susceptibility test and consultation by physician of infection department, we chose the sensitive antimicrobial agents to control the infection. The patient was received vancomycin hydrochloride 800 mg by infusion twice per day in the beginning 4 weeks and linezolid 400 mg orally twice per day in the following 2 weeks. At the same time, the patient was also asked to be immobilized in bed.

**Table 1:** Result of antibiotic susceptibility test

Antimicrobial Agents	Result	MIC (μg/ml)
Ciprofloxacin	Sensitive	≤0.5
Clindamycin	Sensitive	≤0.25
Erythromycin	Sensitive	≤0.25
Gentamicin	Sensitive	≤0.5
Linezolid	Sensitive	≤0.25(2nd)
Oxacillin	Sensitive	≤0.25
Penicillin G	Resistant	≥0.5
Vancomycin	Sensitive	≤0.25(1st)

Antibiotic susceptibility test revealed vancomycin and linezolid were the top two kinds of the most sensitive antimicrobial agents

After 6 weeks conservative treatment, low back pain of this patient was relieved and the patient’s neurological symptoms were recovered. He did not have any points of tenderness on his back, and had 4/5 dorsiflexor strength in his left 1st toe. The straight leg raising test was negative in his bilateral lower extremities. The Visual Analogue Score (VAS) was 2.

**Fig. 3:**
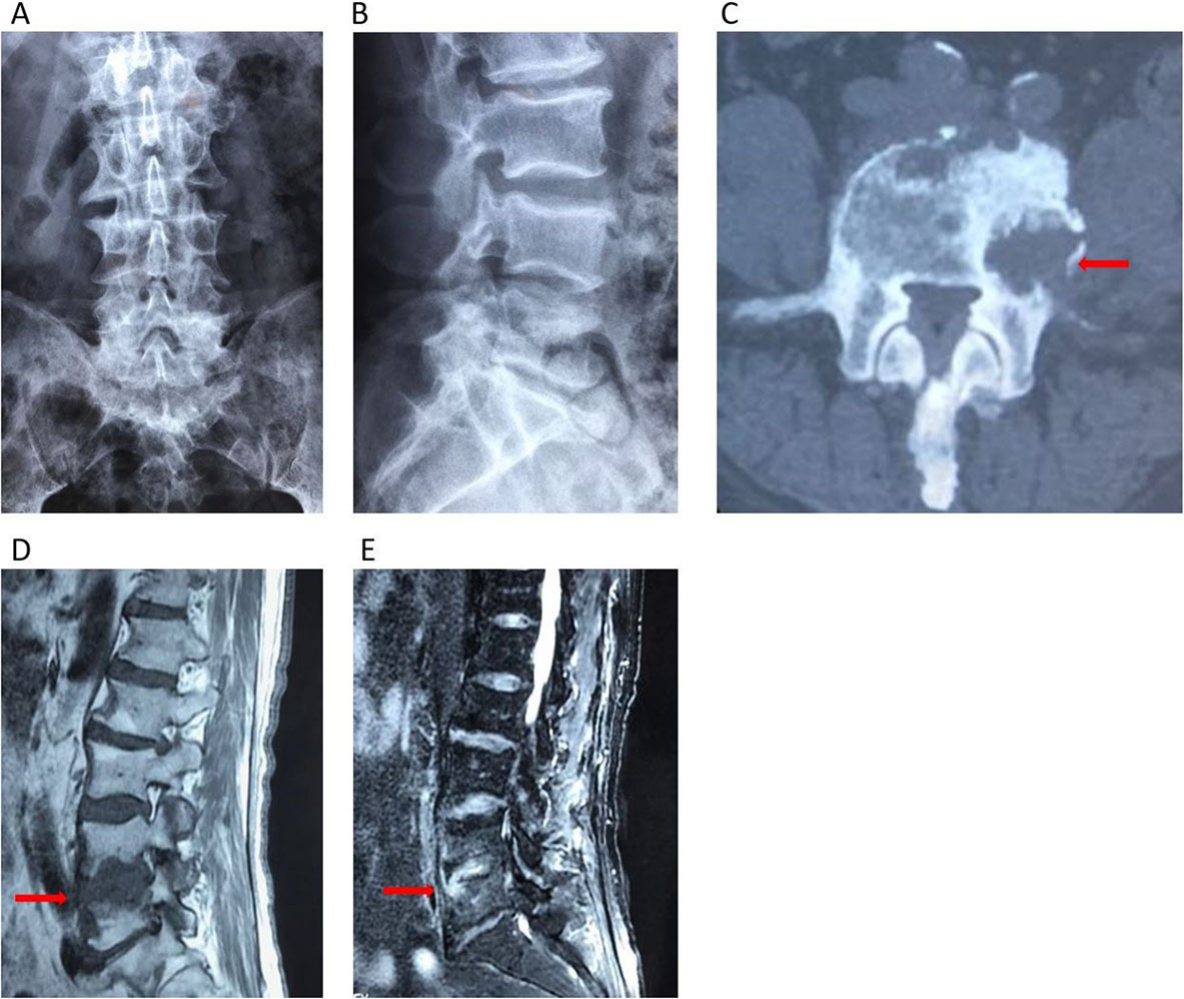
Radiological findings after 6 weeks treatment. Notes: (**a** and **b**) X-ray after treatment showed degeneration of lumbar spine, hyperplasia of vertebrae, no significant narrowing of L4–5 inter-vertebral space**.** (**c**) CT showed the area of vertebral worm-eaten-like damages were reducing and limited, the bone sclerosis appeared around the lesion (arrow).(**d** and **e**) MRI after 6 weeks treatment showed the area of inflammatory signal (hypointense on T1-WI and heterogeneous hyperintense on T2-WI) was significantly reducing (arrow)

**Fig. 4:**
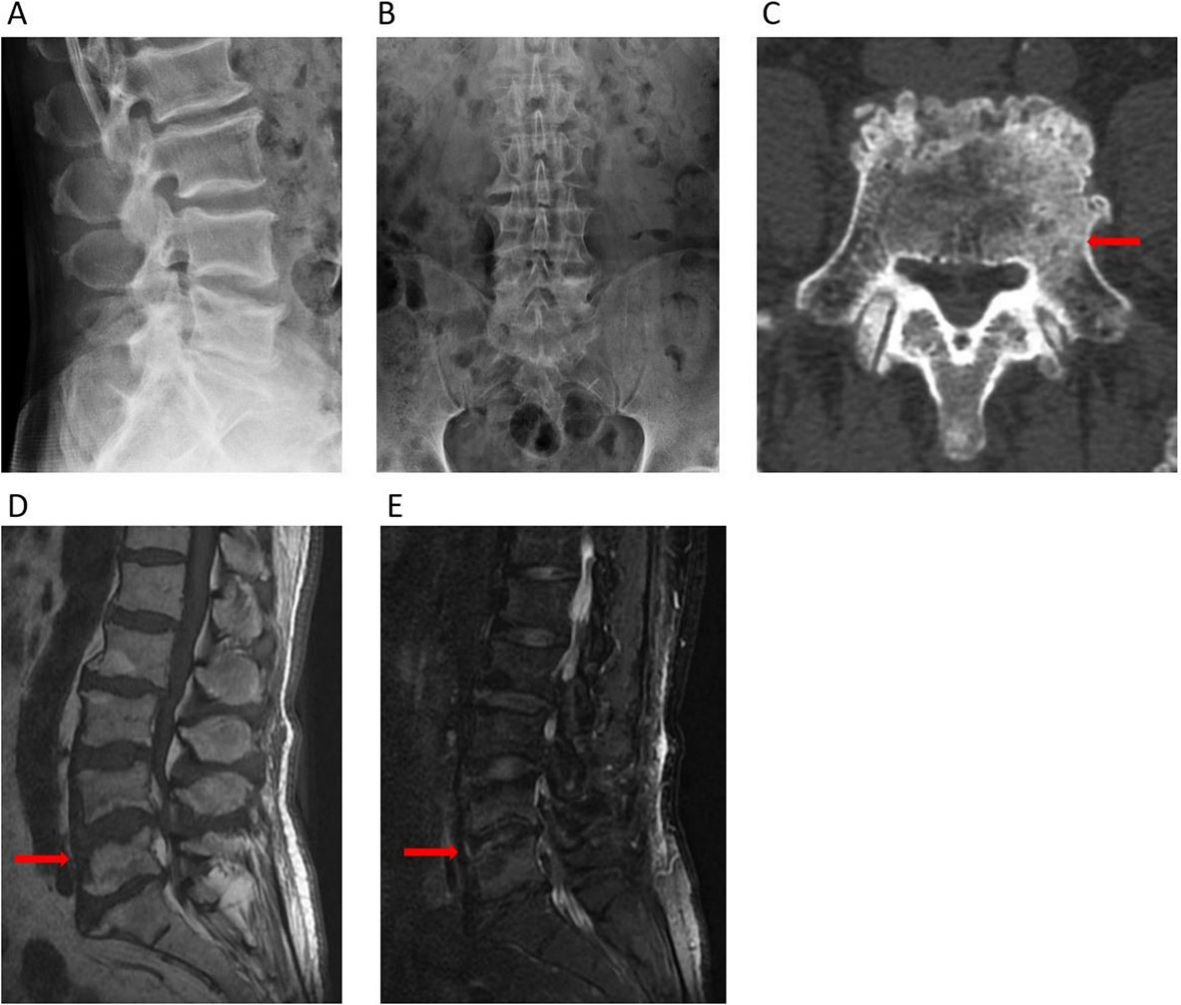
Radiological findings at the 12th month after treatment. Notes: (**a** and **b**) X-ray showed degeneration of lumbar spine, hyperplasia of vertebrae, no significant narrowing of L4–5 inter-vertebral space. (**c**) CT showed the area of vertebral destruction was almost recovered and the bone sclerosis around the lesion (arrow). (**d** and **e**) MRI showed the area of inflammatory signal (hypointense on T1-W images and heterogeneous hyperintense on T2-W images) almost disappeared (arrow)

All of the laboratory test data was within normal limits. Lumbar X-ray showed degeneration of lumbar spine, hyperplasia of vertebrae, no significant narrowing of inter-vertebral space (Fig. 3a and b). CT scan revealed the area of vertebral worm-eaten-like damages were reducing and limited, the bone sclerosis appeared around the lesion (Fig. 3c). MRI of the lumbar spine revealed the area of inflammatory signal (hypointense on T1-WI and hyperintense on T2-WI) was significantly reducing (Fig. 3d and e). After a 12-month follow-up period, X-ray showed no obvious narrowing of inter-vertebral space (Fig. 4a to b). The area of worm-eaten-like vertebral destruction was almost absolutely recovered in CT scan (Fig. 4c). What’s more, MRI indicated inflammatory signal (hypointense on T1-WI and hyperintense on T2-WI) almost disappeared. There was also no symptomatic recurrence or any complaints for this patient, dorsiflexor strength in his left 1st toe was perfectly restored as well.

**Fig. 5:**
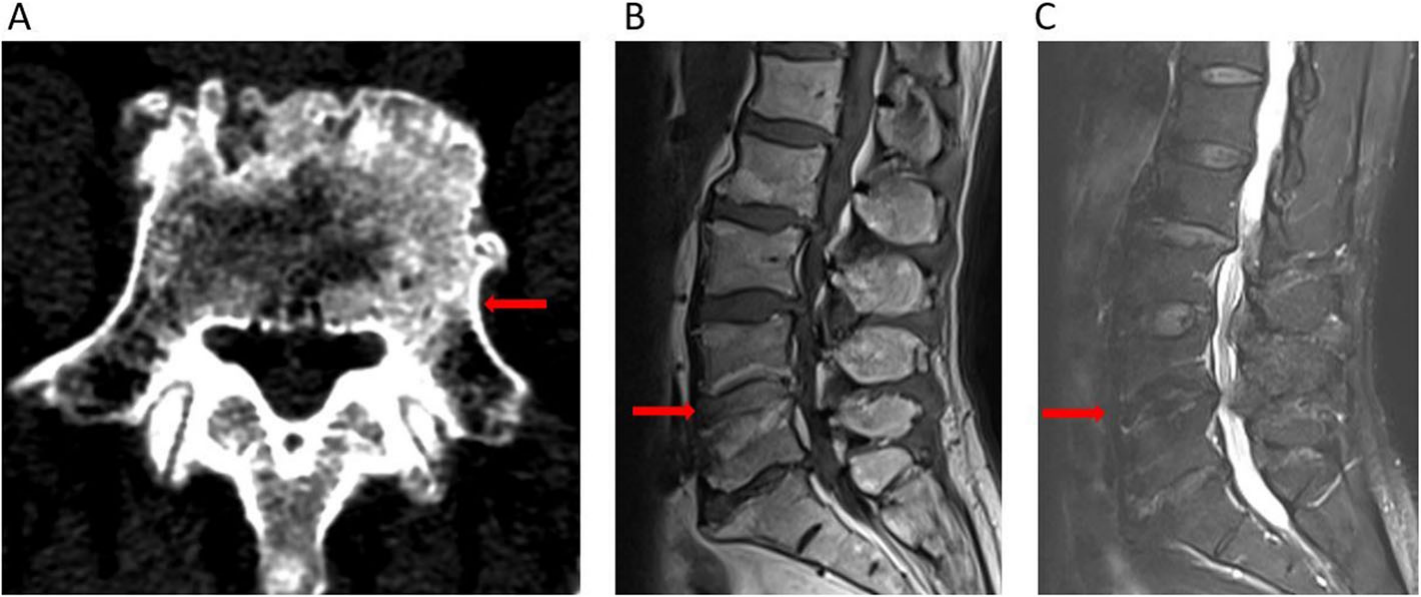
Radiological findings at the 36th month after treatment. Notes: (**a**) CT showed just hyperplasia and deterioration of L5 and no recurrence of vertebral destruction (arrow). (**b** and **c**) MRI showed there was no indication of inflammatory signal (hypointense on T1-WI and heterogeneous hyperintense on T2-WI) (arrow) compared with the images before

The patient came back to hospital for follow-up at the 36th month after treatment with a satisfactory recovery. There wasn’t any recurrence of vertebral destruction in CT (Fig. 5a) or any hints of inflammatory signal in MRI (Fig. 5b and c).

## Discussion and conclusion

Generally, there are three types of Spondylodiscitis recognized. They are pyogenic spondylodiscitis, granulomatous (tuberculous, brucellar, aspergillar, and fungal) spondylodiscitis, and parasitic spondylodiscitis [12]. Pyogenic spondylodiscitis (PS) is a subacute or acute and non-specific spinal infection involving intervertebral discs and adjacent vertebral bodies, which could be potentially intractable or life-threatening in the case of misdiagnosis and delay of appropriate treatment. The mostly infected foci for pyogenic spondylodiscitis is the lumbar spine (58%), followed by the thoracic (30%) spine and cervical spine (11%) [13]. In most cases, PS is known as a hematogenous infection due to causative microorganism disseminated from distant infectious sites. However, in recent years, iatrogenic infections cannot be ignored for the spread invasive diagnostic and surgical procedures, no matter if it is mini-invasive or not. The patient in this case had a history of Traditional Chinese Medicine acupuncture treatment before admitting to hospital, which might be a risk factor of his infection.

The coagulase-negative staphylococci strains are a major cause of nosocomial infections and nonspecific spinal infection [14]. Sc is usually perceived as one of coagulase-negative staphylococcus which are more often associated with non-human animal infection, especially goats [15]. Most infections of Sc were contracted in the hospitals, and a few community-acquired infections have also been reported. Several researchers have reported that Sc is sensitive to some kinds of antibiotics including clindamycin, erythromycin, cephalosporin et al., resistant to methicillin and penicillin [16–18]. Ross [15] reported that Sc with methicillin-resistant was also found in neonatal intensive care unit in which Sc could survive for a long time (28 months or more), they found Sc shared the same characteristic especially in antibiotic profile with Sa as well. Furthermore, biofilm formation and slime production are features of Sc and they may play a crucial role in conferring virulence to this strain [11, 19]. Based on previous studies and reports, we also imitated diagnosis and treatment experience of LPS of Sa in this case.

Patients suffering from LPS usually present unspecific symptoms and signs. Low back pain is most frequently described (85%). Low back tenderness or spasm of paraspinal muscles could be evidently detected in physical examination [20]. Neurological deficits including sensory loss or motor weakness can’t always be found, ranging from 10 to 50% [21, 22]. Fever, an infrequent and unspecific symptom of PS, may sometimes occur [21]. Due to the insidious onset and unspecific presentation of the disease, getting an accurate diagnosis of LPS in the early period is still challenging. Usually a delay of 2 to 6 months has been reported between beginning symptoms and final diagnosis. As a result, the outcome of the treatment is usually poor [23, 24].

Diagnosis should be supported by clinical, laboratory, bacteriological and imaging findings. Though white blood cell (WBC) count is not sensitive and specific enough for the diagnosis of LPS, the increase in WBC count or high proportion of neutrophils is still a very important test for infectious disease [25]. The Elevation of C-reactive protein (CRP) and erythrocyte sedimentation rate (ESR) are also not specific for LPS, but the results of the above two tests are very helpful, with sensitivity of 98 and 100%, respectively [25, 26]. In addition, ESR and CRP are also useful as screening tools and as monitoring standards for response of the treatment. Magnetic resonance imaging (MRI) is considered to be a reliable method to diagnose spinal infection with specificity, sensitivity, and accuracy of over 90% [27] . MRI is also capability to provide details and structures of spine, paraspinal tissues and the epidural space [28, 29]. Hypointense on T1WI and hyperintense on T2WI of vertebral body, disc and paraspinal tissue suggests destruction of endplate, paraspinal tissue and marrow edema of the vertebral body [30].

Computed tomography (CT) is not as specific and sensitive as MRI for early diagnosis of LPS, but it could still be useful in observing the boundary of infected foci and confirming the extent of debridement of infected, necrotic tissues, because MRI may overestimate the infected extent [31]. Three-phase technetium-99 m bone scan is another choice for the diagnosis and estimation of bony infection. Positive results may be detected within a few days after the symptoms onset with the specificity of 78% and sensitivity of 90% [32]. However, the scan may show increased activity for osteoporotic effect and even when LPS is cured with normalization of the laboratory tests [31]. In recent studies, the use of positron emission tomography-computed tomography (PET-CT) is considered to be a promising method in distinguishing tumors, infectious diseases and degenerative changes, as high 18-F Fluorodeoxyglucose (FDG) uptake is seen in spondylitis and tumor [33, 34]. Moreover, PET-CT may also provide detailed analysis of changing in infected lesions over time, and monitoring the response of the treatment [35]. Though PET-CT might be expensive, the appropriate application of it could be highly efficient in identifying diagnosis.

Differential diagnosis from other common pathogens of spinal infection is always needed to be taken into consideration including spinal tuberculosis and brucellosis. Brucellosis is known as a zoonosis caused by small gram-negative non-encapsulated coccobacilli of the genus Brucella. Patients who get infected are often closely contacted with infected animals with poor animal husbandry methods and hygiene standards. The prevalence of musculoskeletal involvement for brucellosis is high, and the most frequent site is spine [36]. Routine laboratory tests such as ESR, full blood counts, and CRP level are non-specific. Serologic tests such as the enzyme-linked immunosorbent assay, counter immune electrophoresis, and the rose Bengal plate test are crucial to establish the disease and define the stage of the disease [37]. Brucella spondylitis is different from other types of spondylitis in that it may appear as either focal or diffuse. In the early stage, there is bone destruction and healing in the superior vertebral endplate, the characterized osteophyte formation is called “parrot’s beak”. On MRI, there is hypointense signal on T1-WI and hyperintense signal on fat-suppressed T2-WI in affected regions [38]. The intervertebral discs aren’t always involved at the early stage. Spinal brucellosis may be similar to other diseases that affect the spine including tuberculous spondylitis, pyogenic spondylitis, intervertebral disc herniation [39]. CT may be helpful in distinguishing other types of spondylodiscitis for the characteristic findings which are destruction of adjacent endplates with disc gas, and a variable amount of bone sclerosis without vertebral collapse, gibbus deformity, or spinal cord compression [40]. Definite diagnosis can be confirmed via isolation of the microorganism from bone marrow, blood or the abscess.

Spine is the most common skeletal site which accounts for about 50% of skeletal tuberculosis (TB). Approximately 50% of all TB patients have a primary lung foci or history of pulmonary TB [35]. Unlike most infections of the spine, 95% of spinal TB begins in the anterior vertebral body and diffuses under the anterior longitudinal ligament involving adjacent vertebral bodies. In contrast to pyogenic spondylodiscitis, the intervertebral disc of spinal TB is often the last to be affected [41]. The tuberculin purified protein derivative (PPD) skin test is usually positive, indicating either past or present exposure to Mycobacterium. Chest x-rays or CT are also important because more than 50% of patients with spinal TB have pulmonary foci [42]. Spinal CT scan detects destruction of bone earlier than plain radiographs. MRI is more crucial and extensively used unless there is a contraindication. In MRI scan, spinal TB appears hypointense on T1WI and hyperintense on T2WI in affected regions. Enhanced MRI can differentiate between degenerative changes and infection. The typical MRI finding is characterized as multiple affected vertebral bodies with well-preserved intervertebral discs [35]. Tubercular paraspinal cold abscesses have a characteristic appearance on MRI which are uniquely distinguishing from pyogenic abscesses. Biopsy of the spine lesion is the gold standard diagnostic test for spinal TB [41]. All tissue samples are suggested to be sent for histopathology, culture and polymerase chain reaction (PCR) [35]. The staining and smear microscopy of acid-fast bacilli (AFB) is also a common method to detect mycobacteria. Cytology shows granulomatous reaction characterized as the gathering of epithelioid cell granulomas, granular necrosis, lymphocytes, and Langerhans giant cells [43].

Since appropriate choice of the antibiotics is almost the most important treatment for LPS, bacteriological proofs of the causative organism are imperative. Although only 30 to 78% of LPS cases are reported to be confirmed by blood cultures, it is still very necessary at the very beginning of the disease regardless of the presence of fever [44]. Once the result of blood cultures is positive, further invasive procedures may not have to be performed. For those patients who are highly suspected for LPS on radiological evaluation with negative blood culture result, biopsy obtained from infected lesion is necessary to confirm the diagnosis. Percutaneous biopsy or puncture is mostly performed with guidance of CT or fluoroscope for the mini-invasive advantage, endoscopic or open surgery may also be done sometimes. Biopsy is considered to have higher overall diagnostic yield in contrast to the various diagnostic methods [44]. In this case, we also irrigated sterile water by syringe through the needle into the lumbar vertebrae and collected the irrigating fluid for culture while doing percutaneous puncture for higher positive possibility. The decision of choosing appropriate antimicrobial agents for the treatment of LPS depends on the identification and drug sensitivity of causative organism obtained by blood or biopsy culture. If pathogenic organism is not found, broad spectrum antibiotics with gram-positive and anti-staphylococcal coverage are recommended [45]. Cefotaxime + Flucloxacillin or Clindamycin +Ciprofloxacin are usually chosen for wide spectrum of potential pathogens [46]. Fortunately, the appropriate antimicrobial agents were soon confirmed with bacterial culture of percutaneous needle biopsy, the patient was cured timely. Whereas, there is yet no randomized controlled trial data regarding the optimal treatment duration, but 4–6 weeks [47], up to 3 months [48] are mostly reported. In this case, systemic antibiotics treatment for the patient was 6 weeks (2 weeks intravenous, followed by 4 weeks oral).

Immobilization is also crucial for the patient’s recovery. It is recommended for those who suffered from severe acute pain to have immobilization in the initial 2 to 4 weeks, and followed by ambulation with appropriate brace [49]. The bracing duration may vary from 3 to 6 weeks, up to 3 months according to the patient’s severity of spinal destruction and deformity [31] .

Though conservative attempt is always the first-line treatment of choice, surgery procedure should be taken into consideration in case that the diagnosis may not be confirmed, poor response to the present treatment is observed, or progressive neurologic deficits and deformity of the spine is noted [50]. Rapid surgical treatment including debridement and stabilization is mandatory, if patients present initially with severe spondylodiscitis, neurologic deficits, progressive septicemia, instability or deformity. Classically, an anterior open debridement has been the preferred treatment with good outcome and low intraoperative complication rates [13], particularly for cervical spondylodiscitis. In some clinical situations, in which an anterior approach is not feasible, posterior transforaminal or posterior approach surgery can be considered, mostly in cases of lumbar discitis or minimal vertebral involvement [51]. However, in some complicated cases with extensive spinal involvement, a combination of anterior and posterior approach is appropriate. Once the open surgical treatment is determined, pathological puncture doesn’t have to be performed, because the affected tissue and pus could be obtained during the debridement for further examination. In recent studies, minimally endoscopic disc debridement could be an alternative to conventional open surgery for single-level LPS for its benefits of less trauma, faster recovery and good clinical results [52, 53].

In conclusion, Sc has been rarely reported as a causative organism of native bone without orthopedic prothesis. Identification of the pathogen and using appropriate antimicrobial agents as soon as possible is still the key point for the treatment on LPS of Sc, especially for the patients with significant comorbidities [54]. To ensure the diagnosis, biopsy or culture from infected tissue is known as the highest diagnostic standard than other methods. For the mini-invasive merit and reliable accuracy, vertebral needle puncture with guidance of CT or fluoroscope has been widely used and recommended at the early stage of LPS. In addition, collecting the irrigating fluid of the potential infected vertebrae during puncture might be a good attempt for higher positive rate of bacterial culture.

## Data Availability

All the data and material are from the patient’s assay and examination of Beijing Friendship hospital, Capital Medical University, which are real, credible and for availability.

## Abbreviations

Sc: Staphylococcus caprae
PS: Pyogenic spondylodiscitis
LPS: Lumbar pyogenic spondylodiscitis
LDH: Lumbar disc herniation
CT: Computed tomography
MRI: Magnetic resonance imaging
PET-CT: Positron emission tomography-computed tomography
